# Difficult Parturition

**Published:** 1874-12

**Authors:** L. B. Anderson

**Affiliations:** Virginia


### DIFFICULT PARTURITION.

BY L. B. ANDERSON, M.D., OF VIRGINIA.

Mrs. A., a delicate, spare, tall lady, was brought to labor in the twentieth year of her age, and the eleventh month after her marriage, on the morning of the 27th September. Pains were irregular, increasing in severity and frequency till six o'clock in the evening, when I was called in. The pains were now quite energetic, and occurring at intervals of five minutes. An examination disclosed the engagement of the head in the upper strait in proper position, with a well developed and capacious pelvis. The pains continued as I found them during the night, and until four o'clock on the evening of the following day, with but slight advance in the labor.

A careful examination of the abdominal parietes exhibited a tumor as large as a well developed fœtal head in the epigastrium, and a slight contraction about the umbilicus, and another tumor, rather larger than the first, extending from the umbilicus to the pubis. The first tumor was as hard and resisting as a fœtal cranium, and produced the impression that there were two children occupying the uterine cavity, with their heads in opposite directions, and that the great elongation of the longitudinal fibres of the uterus had so attenuated them as to weaken their contractile powers to such a degree as to disable them from overcoming the natural obstructions to the passage of the child; while the transverse diameter being less than usual, gave greater vigor to those fibres, and hence the severe but unexpulsive pains. What could be done under the circumstances? Should ergot be given to increase the expulsive throes of the uterus? The pains were already very severe, the passage was ample, and the parts, all, in a laudable state of relaxation. To increase the contractile efforts of the uterus, seemed to promise no special advantage, but rather presaged evil. For the longitudinal fibres could not compete with the transverse, and what would have increased the energy of the one would, in like manner, have increased the other. And as the latter so much preponderated, their increased energy would have retarded the labor in the direct ratio that they excelled the former in capacity. And there was rational ground to fear that such a course might result in rupture of the uterus. I now determined to lessen the distension of the uterus as much as possible, by rupturing the membranes and permitting the escape of the liquor amnii. But the membranes were tight, drawn over the head of the child, and when ruptured, not more than a spoonful of fluid exuded. The pains were now very severe, the superior portion of the occipital bone wast resting against the pubis, and the whole energy of the uterus seemed to be expended in its transverse contractions. I determined, therefore, with every pain, to endeavor to depress the head with the finger of one hand, and with the palm of the other to give gentle but firm pressure to the superior tumor downwards. Each succeeding pain caused the expulsion of a little water, and every effort to move the tumor downwards resulted in bringing it in nearer proximity to the pubis—in lessening its longitudinal diameter—in obliterating the hour-glass contraction, and giving rotundity and expulsive power to the wound. This was done with every pain, and to my gratification, I perceived the labor was advanced by every pain, and at six o'clock P. M. of the second day, she was delivered of a large and living child.

The sequel demonstrated the fact, that the epigastric tumor was produced by a rigid contraction of the uterus upon an adherent placenta, and the feet of the child—the depression was caused by a firm transverse contraction upon the lower extremities just below the pelvis. And the child occupied a vertical position, with the head downwards, and while it was engaged in the pelvic passages, the body was perfectly straight, the lower limbs fully extended, and the feet and placenta occupied the epigastric region. No means, of which I can conceive, would have promised such success as those described above. And without the artificial mechanical aid afforded the longitudinal fibres, I can't see how delivery could have been effected. The cord was wrapped around the neck, and assisted in retarding the last expulsive movements. There was some difficulty in detaching the placenta, and there was more than usual hemorrhage, which was easily controlled by mechanical irritation, producing uterine contraction. She has since done well.

